# Global carbon sequestration through continental chemical weathering in a climatic change context

**DOI:** 10.1038/s41598-021-02891-y

**Published:** 2021-12-08

**Authors:** Juan Luis Lechuga-Crespo, Sabine Sauvage, Estilita Ruiz-Romera, Michelle T. H. van Vliet, Jean-Luc Probst, Clément Fabre, José Miguel Sánchez-Pérez

**Affiliations:** 1 Department of Chemical and Environmental Engineering, University of the Basque Country, Plaza Ingeniero Torres Quevedo 1, 48013 Bilbao, Basque Country Spain; 2 Laboratoire Ecologie Fonctionnelle et Environnement, CNRS, UPS, Toulouse INPT, Université de Toulouse, Campus ENSAT, Avenue de l’Agrobiopole, 31326 Castanet Tolosan Cedex, France; 3 Department of Physical Geography, Faculty of Geosciences, Utrecht University, P.O. Box 80115, 3508CB Utrecht, The Netherlands; 4 Eawag: Swiss Federal Institute of Aquatic Science and Technology, Dübendorf, Switzerland

**Keywords:** Biogeochemistry, Element cycles

## Abstract

This study simulates carbon dioxide (CO_2_) sequestration in 300 major world river basins (about 70% of global surface area) through carbonates dissolution and silicate hydrolysis. For each river basin, the daily timescale impacts under the RCP 2.6 and RCP 8.5 climate scenarios were assessed relative to a historical baseline (1969–1999) using a cascade of models accounting for the hydrological evolution under climate change scenarios. Here we show that the global temporal evolution of the CO_2_ uptake presents a general increase in the annual amount of CO_2_ consumed from 0.247 ± 0.045 Pg C year^−1^ to 0.261 and 0.273 ± 0.054 Pg C year^−1^, respectively for RCP 2.6 and RCP 8.5. Despite showing a general increase in the global daily carbon sequestration, both climate scenarios show a decrease between June and August. Such projected changes have been mapped and evaluated against changes in hydrology, identifying hot spots and moments for the annual and seasonal periods.

## Introduction

Chemical weathering of rocks has a significant impact on long-term global climate regulation^1^. It transforms soil CO_2_ into inorganic dissolved carbon (such as HCO_3_^−^ and CO_3_^2−^), which is later exported by rivers to other water bodies^2–11^. Riverine dissolved loadings may be used as a proxy for chemical weathering assessment^4,11^, and it has been recognised that hydrology impacts these flux-discharge relationships^5–7^. Nevertheless, it is unclear how the potential impacts of climate change will affect these dissolved mass balances. Forecasting how potential short-term (daily or monthly) shifts in hydrology under a changing climate may alter these fluxes of riverine matter is needed to assess the potential evolution of the global carbon cycle under a wide range of future scenarios. Here we hypothesized that a more significant seasonality of the hydrological cycle would cause a heterogeneous spatial and temporal pattern in CO_2_ sequestration at the global scale.

Climate change scenarios show increases in air temperatures and significant changes in the hydrological cycle^12^. Still, its effect on chemical weathering and related CO_2_ uptake dynamics and dissolved solids exportation by rivers is poorly understood^13^. For instance, on one side, an increase in the soils microbial activity is expected, implying an increase in CO_2_ production through respiration^14,15^. However, higher air temperatures may decrease CO_2_ dissolution in water, as carbonate weathering follows a boomerang-shaped evolution with increasing temperature^16^. Hydrology is the dominant driver of matter transport at large scale^17^. Nevertheless, the potential implication of respiration and temperature in the global balance remains poorly understood^13^.

How will the annual soil CO_2_ consumption evolve under these hydrological shifts? Where and when will these soil CO_2_ consumption changes be more relevant? The answer to these scientific questions needs a large-scale comprehensive field study. However, such a study is challenging regarding the in situ resources needed to measure the variables involved in these biogeochemical cycles.

In this complex framework, modelling arises as an alternative approach to overcome this challenge by yielding insights into where and when hot spots and hot moments (places and times with disproportionally high chemical weathering rates) may be located. Identifying potential hot spots and moments of change is important to target areas needing the deployment of resources to understand in situ processes, which could yield relevant insights for landscape biogeochemistry. Two main types of models exist for our approach: mechanistic models if processes are constrained by basic physical principles, such as thermodynamic laws and conservation of mass, and empirical models when built using parametric relationships derived from observational data. Mechanistic models, such as B-WITCH^15,18^ and RT-Flux-PIHM^19,20^, link thermodynamics, kinetics and transport processes to hydrology and vegetation. They have yielded insights, for instance, that 40% of the total increase in CO_2_ consumption in the Mackenzie River basin is related to the direct effect of climate change on hydrology alteration^15^. Moreover, they highlighted that a “chemostatic” behaviour of riverine dissolved load suggests that hydrological changes impact the dissolved element concentration in rivers^19,20^. Empirical models are commonly developed at large spatial scales^7,21^, and their results are used in relevant assessments such as the ESCOBA program^22^ and the IPCC Assessment Reports^12^.

The objective of this study was to assess the impact of future modifications of the hydrological cycle on the global CO_2_ sequestration by weathering. Based on the largest basins in the world, we aimed at identifying the watersheds or areas with higher CO_2_ consumption compared to the rest of the globe (hot spots^23^) and the periods showing larger sequestration (hot moments^23^) on which to focus research efforts.

In this study, a cascade of models was set up to cover the 300 largest basins in the world, obtaining the spatial and temporal potential evolution of CO_2_ sequestration under two climate change scenarios (RCP 2.6 and RCP 8.5). Historical and future dynamics of cations and anions derived from chemical weathering of rocks and associated carbon sequestration were modelled by coupling the hydrological model VIC with the geochemical model ICWR. Then, the results from this set-up were used as an input in the MEGA model to estimate CO_2_ consumption. Observed data have been used to evaluate the results at each step of the model cascade. The reader is referred to the methods section and the Extended Data Fig. 2.

## Results and discussion

### Interannual and seasonal fluctuations

**Figure 1:**
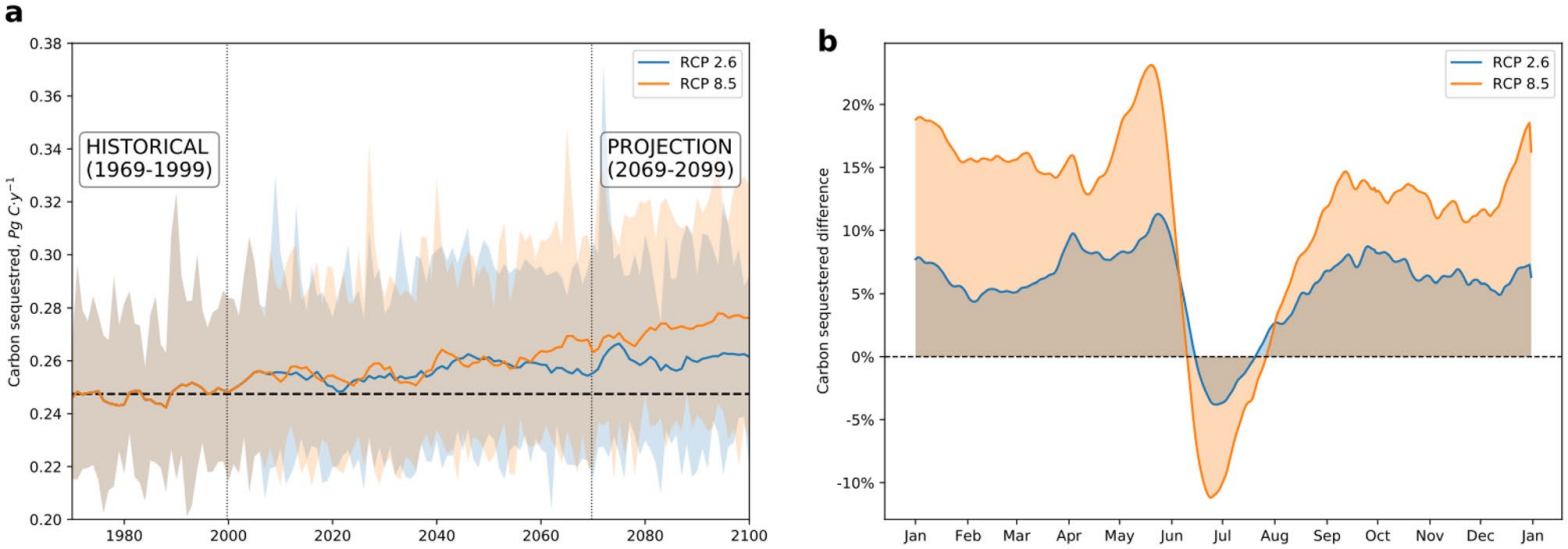
Temporal evolution of the global soil CO_2_ sequestered for the RCP 2.6 (blue) and RCP 8.5 (orange) scenario: **(a)** interannual fluctuations in annual weathering CO_2_ consumption, where shaded areas represent the minimum and maximum amount of annual carbon sequestered in five general circulation models, and the solid lines account for a 10-year moving average to evaluate the pattern. The dashed horizontal line represents the mean consumption for the Historical period. **(b)** Seasonal variations for the daily mean relative difference between the Projection (ECP) and Historical periods.

The annual mean inorganic C sequestration through chemical weathering during the Historical period (1969–1999) amounts to 0.247 Pg C year^−1^ (1 Pg = 10^15^ g), in the lower range of previous research^4,8–11,21,24–27^, spanning from 0.22 to 0.30 Pg C year^−1^. The climate change scenarios present a potential increase in the amount of C sequestered, reaching mean values of 0.261 and 0.273 Pg C year^−1^ for RCP 2.6 and RCP 8.5, respectively. The annual series (Fig. 1a) shows a similar temporal evolution for the middle part of the century, but such a difference enlarges within the “End of Century Projection” (ECP) period (2069–2099). An annual average increase in C sequestration of 6% (RCP 2.6) and 10% (RCP 8.5) is forecasted if the Historical period is considered the baseline. The annual increases represent < 0.1% of the anthropogenic emissions projected for 2100 in the RCP 8.5 scenario and ~ 0.7% of the "negative emissions" projected for the RCP2.6 scenario^28^. These C sequestration increases are low at the global scale; however, these become more relevant when evaluating the change percentages at a lower time-step (daily).

When comparing the mean daily temporal evolution in the ECP and the Historical periods (Fig. 1b), more considerable differences are found for the RCP 8.5 scenario. In both scenarios, a positive difference is found for most of the year, agreeing with a more considerable amount of C sequestered during the ECP period compared to the Historical one. Such increases may reach a positive 20% value in May for the RCP 8.5 scenario, followed by a shift towards a decrease between June and August. It suggests a lower CO_2_ consumption through continental chemical weathering during these months. Previous studies highlighted that hydrology is a dominant driver in the temporal evolution of CO_2_ consumption^29^. Therefore, a decrease in CO_2_ consumption suggests that a lower amount of water is being exported in this period. This decrease coincides with the low discharge period in the northern latitudes, suggesting that these regions significantly influence the global amount of soil CO_2_ consumed through chemical weathering. The Northern Hemisphere has a more considerable amount of continental land and the hydrological cycle is expected to intensify in these areas. This suggests that the annual shift found between June and August regarding the global carbon weathering CO_2_ uptake could be a consequence of hydrological changes at these latitudes.

### Hot spots and moments for CO_2_ uptake

**Figure 2:**
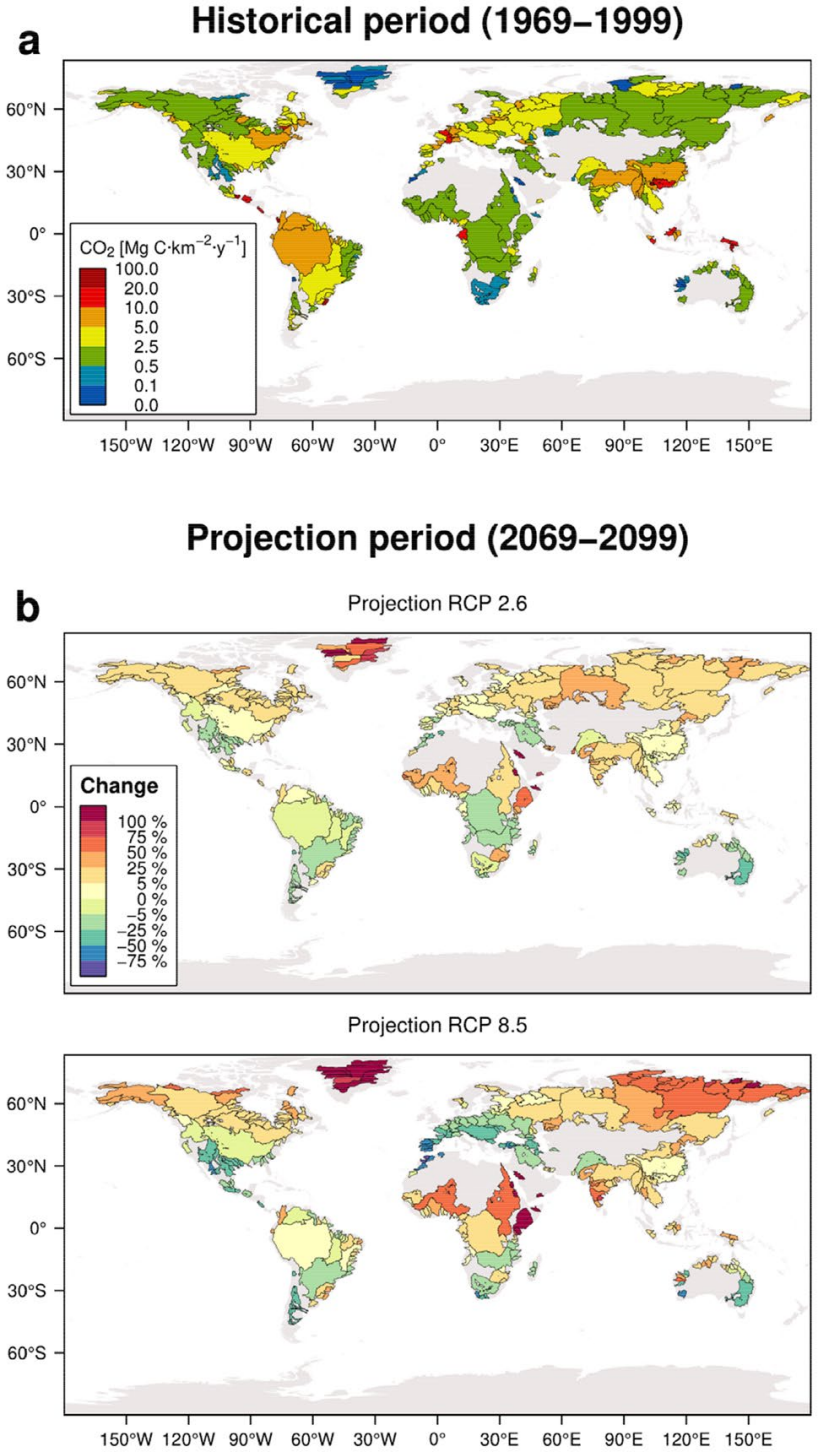
**(a)** Interannual mean CO_2_ consumption through chemical weathering during the Historical period, expressed as Mg C year^−1^ km^−2^; and **(b)** relative change for the Projection (ECP) period in the RCP 2.6 and RCP 8.5 climate scenarios, expressed as a percentage of change regarding the Historical period. The maps were created with the R software (version 4.0.3; https://www.r-project.org/).

On a global scale, the spatial distribution of carbon sequestration during the Historical period (Fig. 2a) is comparable to what is described in previous literature ^7^. Similarly, values are analogous to regional cases, such as the Amazon^11,29,30^, Congo^5,21^, Niger^31^, Garonne^21^, the Alps region^32^ or the United States continuum^33^, even if the lateritic soils have lower CO_2_ uptake, as shown by Boeglin & Probst^31^.

Alterations in this spatial pattern (Fig. 2b) are considered primary consequences of hydrological changes since the geochemical processes take place over a much larger time scale than biogeochemical processes on the Earth surface. More significant changes are found in the northern latitudes, where an increase in the annual discharges in these river basins is expected due to a larger precipitation amount, an earlier onset and a more intensive snowmelt in spring^34^. Even though a higher seasonality will change the hydrological cycle within the year.

A general increasing trend in CO_2_ sequestration is found during the January–February–March (JFM) period (Extended Data Fig. 1), especially in basins such as the Yenisei and the Ob, which present changes > 25%, particularly in the RCP 8.5 scenario. Increasing temperatures are expected to cause an accelerated snowmelt^34^, which could lead to the increase of dissolved inorganic carbon river fluxes. In contrast, heterogeneous projections are found for southern latitudes, as in the Murray-Darling River basin in Australia and the Orange River in South Africa. These basins are located under mixed climatic zones^35^, which could explain the heterogeneous results found in the scenarios. The potential evolution in the river discharge seasonality in these areas may cause different geochemical fluxes since a discharge decrease of more than 25% in the low-flow period is expected^34^. However, further analysis using mechanistic approaches or in situ measurements is needed to understand and assess potential changes in biogeochemical cycles in these regions during the January to March (JFM) period.

A different pattern is found for the July–August–September (JAS) season, when most of the northern latitudes present negative changes, suggesting a decrease in the CO_2_ uptake due to chemical weathering. This shift in the northern latitudes is attributed to a projected decline in discharge, notably on the RCP 8.5 scenario^36^.

**Figure 3:**
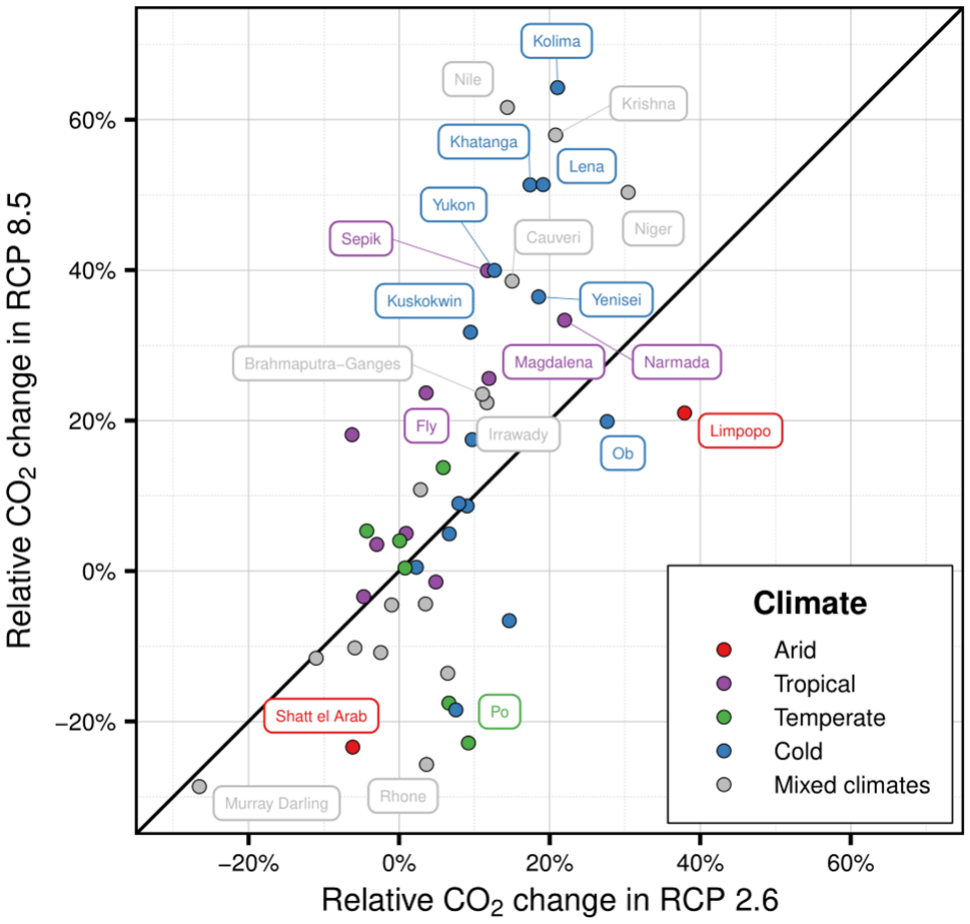
Comparison of the relative CO_2_ uptake change in the river basins used for MEGA^21^ model validation for both climatic scenarios. Only river basins showing a relative change over ± 20% are labelled. The straight black line represents equal change.

As discharge intensity is a primary critical factor of CO_2_ flux changes in the short-term (daily, monthly or seasonal) assessment, we compared the relative changes in CO_2_ uptake in a subset of river basins for both scenarios (Fig. 3). Such a comparison illustrates how watersheds located in tropical and cold climates present significant differences for CO_2_ uptake, especially basins in the Siberian region (e.g. Kolyma, Khatanga or Lena) or active arc-islands in New Guinea (e.g. Fly or Sepik). These changes show that, even with their smaller draining areas and discharges, the role of arc-islands should be taken into consideration and should be a focus of future research as their relative role in carbon sequestration through chemical weathering is relevant^7^. Besides, the potential impacts of climate change in these tropical areas are expected to be more relevant in these smaller river basins than in larger ones like the Amazon or Congo^7^.

Similarly, the relative change in CO_2_ sequestration on watersheds with mixed climates (e.g. the Nile, Krishna, Niger or Murray Darling) is large. It could be related to higher amounts of carbonate rock outcrops in these areas, releasing significant amounts of CO_2_ during weathering^37^. Regarding cold and polar basins, permafrost processes were not included in the present analysis. They are expected to significantly impact the carbon sequestration by weathering and the loads to rivers^38^.

Even though there are some relative changes noted at the annual scale, these differences are not maintained throughout the year. The short-term temporal evolution should be a focus of interest for future research, especially in those areas located in tropical and cold climates since they seem to be more sensitive to climate change.

### Modelling approach limitations

This study uses a model output as input to another model, which implies a propagation of the error in each step. We assessed the uncertainties of our approach with previously published data. The results show a very significant correlation to observed data^27^ for the ICWR model (ρ_SPEARMAN_ = 0.83, p < 0.01, n = 173, see Extended Data Fig. 5), as well as for the MEGA model results in the Historical period^6^ (ρ_SPEARMAN_ = 0.71, p < 0.01, n = 48, see Extended Data Fig. 6). In summary, even though a cascade of models usually accumulates errors, the present modelling approach fits well with current observed data.

Continental weathering occurs at the critical zone, where slow geological processes interact with faster biogeochemical ones. The present modelling approach includes a macroscale hydrological model, which resolves the energy and water balance. The main uncertainties are discussed elsewhere^36^. Concerning the ICWR model, the parameters have been fitted to observed data for a given situation. These laws may change under a climate change scenario. The MEGA model balance is based on a set of hypotheses described in the literature and summarised in the Extended Data Fig. 4. The main limitation of this modelling set-up is that it does not simulate transient states, as the chemical weathering rates are described through linear equations. Regolith thickness is kept constant during simulation even though it is coupled to physical erosion, which could change chemical weathering rates^39^. Temporal variation of below-ground CO_2_ concentration is not included in the ICWR model, even though it has been noted as relevant in silicate weathering and affected by vegetation changes^40^. Further uncertainties are related to the resolution of the lithological map (see “Methods” section). It has already been shown that using a map with a finer resolution helped improve the modelling results at local scale^41^.

## Conclusion

This study presents a forecast of the potential evolution of CO_2_ consumption during the chemical weathering of continental rocks at the global scale under two climate change scenarios. The research provides two main new insights on the temporal evolution of the carbon cycle in the short-term, derived from shifts in the water cycle: (a) Even though there is a trend towards increasing the amount of carbon sequestered at the annual and global scale, this pattern is not homogeneously distributed neither in space nor time, and (b) the river basins located in cold climates (such as the Kolyma, Lena, Yukon, Kuskokwim or Yenisei) and in tropical climates with a lower draining area (like the Fly, Sepik or Narmada) may be more sensitive to the effects of climate change.

The insights presented suggest that research on the terrestrial part of the carbon cycle should focus on these areas to constrain the main cycle-shaping drivers better, targeting an understanding of the carbon fluxes’ temporal dynamics. This study provides the first snapshot of carbon sequestration’s potential evolution through the chemical weathering of rocks on a global scale, which may be used for comparison in future studies in this field. Even if the carbon fluxes from chemical weathering are small compared to the total CO2 emissions, they are in the same order of magnitude with the natural CO2 emissions to the atmosphere. Local increases in chemical weathering could lead to acidification of local surface waters, which could affect the equilibrium of freshwater and coastal ecosystems.

## Methods

Four Representative Concentration Pathways (RCP) were selected by the Intergovernmental Panel on Climate Change (IPCC) in its fifth Assessment Report^12^ regarding climate change impacts on the carbon cycle. Two of those four RCPs are included in the present analysis (RCP 2.6 and RCP 8.5). RCP 2.6 considers that the radiative forcing (i.e. the strength of change drivers) will peak at about 2.6 W m^−2^ in 2100 and then decline, whereas RCP 8.5 considers that the radiative forcing will reach > 8.5 W m^−2^ by 2100. The highest and lowest RCPs were selected to capture the broadest range of future expected atmospheric greenhouse gas concentrations. Bias-corrected climate model outputs of five general circulation models (GCM) were included in the VIC hydrological model to simulate impacts on daily river discharge on 0.5° × 0.5° at the global scale^36^: GFDL-ESM2M^42,43^, HadGEM2-ES^44,45^, IPSL-CM5A-LR^46^, MIROC-ESM-CHEM^47^, and NorESM1-M^48^. Validation results showed that the observed hydrological conditions were realistically represented by the VIC hydrological model for most river basins. The average daily discharges from the five GCM and its respective uncertainty were taken as an input for the geochemical model.

The input data handling and modelling in the present study follow the workflow summarised in Extended Data Fig. 2. First, a subset of the 300 largest river basins covering ~ 70% of the global land area was selected from the HydroBASINS dataset^49^, including only exoreic river basins (Extended Data Fig. 3). The catchment area selected was derived from the HydroBASINS dataset. Second, the outlet points of river basins from the simulated river discharge VIC database were selected manually by exploring the time series for each sampling location and the surrounding points (± 0.5°) to choose the correct outlet. When a catchment presented several outlets (especially for endorheic catchments draining to a lake or inner sea), all of them were considered, and their discharge were added to account for all water flowing out of the drained area. Deconvolution of the daily discharge signal for each outlet is applied to distinguish between the surface runoff from the soil and groundwater flow. Third, draining area characteristics are summarised for each river basin by considering the relative abundance of lithological classes contained in the Global Lithological Map^50^ (GLIM), the soil classes as defined by the Harmonized World Soil Database^51^ (HWSD), and the climatic zones present according to the Köppen-Geiger classification presented by Beck et al.^35^. The modelling approach consists of two models in cascade; the flux of ions derived from the chemical weathering of rocks is computed using the ICWR^17^ model at a daily time step for each river basin under each discharge time series. Then, the Major Element Geochemical Approach^37^ (MEGA) model uses these loadings to estimate the CO_2_ consumed through chemical weathering.

From this set-up, a daily time series of major ion river fluxes and CO_2_ consumption through chemical weathering from the 300 largest river basins were obtained from 1965 to 2100. Two periods are established to estimate the potential effect of hydrological shifts on weathering CO_2_ consumption: the Historical (October 1969-September 1999) and the ECP (October 2069-September 2099). A comparison between the two periods is also accomplished. Daily discharge time series have been taken from all these scenario-model combinations while temporal aggregation is performed after applying the ICWR and MEGA models.

The stream discharge time series have been deconvoluted to separate the influence of the surface runoff from the baseflow and interflow discharges. Such deconvolution is performed following the digital filter shown in Eq. (1), as proposed by Eckhardt^52^. In the present study, only interflow and baseflow are considered significantly transporting ions, while surface runoff is the principal agent for dilution. A digital filter based on the slopes of the increasing and decreasing parts of the hydrograph was selected. It is sensitive to changes in the amount of water and seasonality and flood events. Nonetheless, this is a source of uncertainty as the infiltration process depends on catchment-to-catchment properties, while this equation assumes linearity between the groundwater outflow (baseflow) and its storage^52^.

1 $$\mathrm{b}\left(\mathrm{t}\right)=\frac{\left(1-{\mathrm{BFI}}_{\mathrm{mx}}\right)\cdot \mathrm{a}\cdot \mathrm{b}\left(\mathrm{t}-1\right)+\left(1-\mathrm{a}\right)\cdot {\mathrm{BFI}}_{\mathrm{mx}}\cdot \mathrm{Q}\left(\mathrm{t}\right)}{1-\mathrm{a}\cdot {\mathrm{BFI}}_{\mathrm{mx}}},\quad \mathrm{ for\,\,\,\, b}\left(\mathrm{t}\right)<\mathrm{Q}(\mathrm{t})$$

where *b(t)* is the baseflow time series, *BFI*_*mx*_ represents the long-term ratio of baseflow to total streamflow, *a* is the filter parameter, and *Q(t)* is the total discharge. When *b(t)* > *Q(t)*, then *b(t)* is replaced by *Q(t)*. The parameters that were selected for the separation were *BFI*_*mx*_ = *0.8* and *a* = *0.95* (as recommended by Xie et al.^53^). These parameters were kept constant in all catchments, which also became a source of uncertainty.

The ICWR model is an empirical model developed to estimate major ion riverine fluxes released by chemical weathering of rocks at the global scale, based on Eq. (2). It has been validated at the global scale under static conditions^17^ and at the local scale under dynamic evolution^41^. Computing it requires a description of the draining area of each river basin in terms of soil cover and lithological distribution. The parameters of the equation take into account the concentration for each ion *x* drained from a lithological class *i*. The ICWR model parameters were calibrated after an atmospheric input correction. Thus, the loadings computed from this equation only relate to the chemical weathering process, which is needed as an input for the MEGA model.

2 $${\text{F}}_{{\text{x}}} \left( {\text{t}} \right) \, = {\text{ b}}\left( {\text{t}} \right) \, \cdot{\text{ f}}_{{{\text{sx}}}} \cdot\sum \left( {{\text{L}}_{{\text{i}}} \cdot{\text{ C}}_{{{\text{ix}}}} } \right)$$

where *F*_*x*_ represents the specific flux of ion *x* in mol km^−2^ year^−1^, *b* represents the baseflow and interflow obtained from the deconvolution of the total discharge signal, *f*_*sx*_ is a factor considering the soil shielding effect on CW (adim), *L*_*i*_ is the relative abundance of a lithological class *i* in terms of the total area of the drainage basin (expressed in the 0–1 range), and *C*_*ix*_ is the calibrated parameter that represents the concentration of the ion *x* draining from the lithological class (expressed in mol L^−1^). This equation is applied to the time series of the deconvoluted discharge, including the baseflow and interflow.

The MEGA model is based on the mass balance shown in Extended Data Fig. 4^37^. The input data are the riverine molar loadings of each major ion, after the atmospheric input correction. We assume that anthropogenic influence is not captured in the measurements of major ion concentrations. *R*_*pyr*_ and *R*_*sil*_ molar ratios for each catchment account for the Ca^2+^ + Mg^2+^ loads coming from the weathering of silicate rocks and the SO_4_^2−^ originating from pyrite oxidation, respectively. The results from this model relate to the CO_2_ consumed by the CW of rocks.

Considering the river loadings of all major ions, the CO_2_ consumed is computed as follows: first, the atmospheric deposition (wet and dry) must be removed from these loads, remaining the molar fluxes derived from CW. All Cl^−^ is associated with the dissolution of halite (NaCl), though the remaining Na^+^ is associated with Na-silicates. If the Cl^−^ > Na^+^, the remaining Cl^−^ is linked to the dissolution of sylvite (KCl). Evaporites do not uptake CO_2_ when dissolving, while Na^+^ and K^−^ silicate rocks (e.g. albite NaAl_3_O_8_ and orthoses KAlSi_3_O_8_) require 1 mol CO_2_ for each ion mol released to water. Then, using *R*_*pyr*_, it is possible to discriminate the SO_4_^2−^ load released respectively by gypsum (CaSO_4_) dissolution and pyrite (FeS_2_) oxidation. Gypsum does not consume CO_2_ during dissolution, while pyrite releases 2 mol CO_2_ for each SO_4_^2−^ ion released. Later, the *C*_*b*_ (molar flux of Ca^2+^ and Mg^2+^ released by carbonates dissolution) is computed using *R*_*sil*_ to account for the quantity of Ca^2+^ and Mg^2+^ derived from the weathering of the carbonates; a 60% of Ca^2+^ is linked to calcite (CaCO_3_) dissolution while the remaining 40% is released by dolomite (CaMgCO_3_) dissolution. The dissolution of calcite and dolomite also takes up to 1 mol of CO_2_. The remaining Ca^2+^ and Mg^2+^ fluxes are linked to silicates that consume 2 mol CO_2_. A further description of the model calculation is found in Amiotte-Suchet^54^, Amiotte Suchet and Probst^37^ and Donnini et al.^32^. The *R*_*pyr*_ and *R*_*sil*_ values used in the present study are compiled in the Extended Data Table 1.

Two 30-year periods are considered in the present study: a Historical (1969–1999) and a ECP (2069–2099), both computed at the daily time scale. The potential impacts derived from climate change are computed by quantifying the relative difference in the ECP period to the Historical period, following Eq. (3). Where *ΔCO*_*2*_ denotes the relative change for each basin (expressed in %), and *L*_*CO2*_ represents the mean amount of CO_2_ consumed through CW of rocks during the ECP and Historical period, expressed in Mg C year^−1^. This result analysis is divided into two steps: at the annual scale, where *L*_*CO2*_ is the mean annual amount of CO_2_ consumed in each period; and at the season scale, where *L*_*CO2*_ symbolises the mean amount of CO_2_ consumed during all the springs, summers, autumns, and winters of each period.

3 $$\mathrm{\Delta C}{\mathrm{O}}_{2}\left(\mathrm{\%}\right)=\frac{\left({\mathrm{L}}_{{\mathrm{CO}}_{2}}^{\mathrm{ECP}}-{\mathrm{L}}_{{\mathrm{CO}}_{2}}^{\mathrm{Historical}}\right)}{{\mathrm{L}}_{{\mathrm{CO}}_{2}}^{\mathrm{Historical}}}\cdot 100$$

## Supplementary Information

Supplementary Information.

## Data Availability

The data that support the findings of this study are available from the corresponding author upon reasonable request.

## References

[1] Calabrese S, Parolari AJ, Porporato A (2017). Hydrologic transport of dissolved inorganic carbon and its control on chemical weathering. J. Geophys. Res. Earth Surf..

[2] AmiotteSuchet P, Probst JL (1993). Modelling of atmospheric CO2 consumption by chemical weathering of rocks: Application to the Garonne, Congo and Amazon basins. Chem. Geol..

[3] Amiotte Suchet P, Probst JL, Ludwig W (2003). Worldwide distribution of continental rock lithology: Implications for the atmospheric/soil CO2 uptake by continental weathering and alkalinity river transport to the oceans. Global Biogeochem. Cycles..

[4] Bhatt MP, Hartmann J, Acevedo MF (2018). Seasonal variations of biogeochemical matter export along the Langtang-Narayani river system in central Himalaya. Geochim. Cosmochim. Acta.

[5] Dupré B (2003). Rivers, chemical weathering and Earth's climate. C.R. Geosci..

[6] Gaillardet J, Dupré B, Louvat P, Allègre CJ (1999). Global silicate weathering and CO2 consumption rates deduced from the chemistry of large rivers. Chem. Geol..

[7] Hartmann J, Jansen N, Dürr HH, Kempe S, Köhler P (2009). Global CO2-consumption by chemical weathering: What is the contribution of highly active weathering regions?. Global Planet. Change.

[8] Probst JL, Ludwig W, AmiotteSuchet P (1997). Global modeling of CO2 uptake by continental erosion and of carbon river transport to the oceans/Modélisation à l'échelle globale des flux de CO2 consommé par l'érosion continentale et des transports fluviaux de carbone vers les océans. sgeol.

[9] Probst JL, Mortatti J, Tardy Y (1994). Carbon river fluxes and weathering CO2 consumption in the Congo and Amazon river basins. Appl. Geochem..

[10] Dessert C, Dupré B, Gaillardet J, François LM, Allègre CJ (2003). Basalt weathering laws and the impact of basalt weathering on the global carbon cycle. Chem. Geol..

[11] Oliva P, Villa IM, Dupré B (2003). Chemical weathering in granitic environments. Chem. Geol..

[12] Ciais, P. *et al.* Carbon and other biogeochemical cycles. in *Climate Change 2013: The Physical Science Basis. Contribution of Working Group I to the Fifth Assessment Report of the Intergovernmental Panel on Climate Change,* edited by T. F. Stocker*, et al.* (Cambridge, 2013).

[13] Liu Z, Dreybrodt W, Wang H (2010). A new direction in effective accounting for the atmospheric CO2 budget: Considering the combined action of carbonate dissolution, the global water cycle and photosynthetic uptake of DIC by aquatic organisms. Earth Sci. Rev..

[14] Beaulieu E, Goddéris Y, Donnadieu Y, Labat D, Roelandt C (2012). High sensitivity of the continental-weathering carbon dioxide sink to future climate change. Nat. Clim. Change.

[15] Conant RT (2011). Temperature and soil organic matter decomposition rates - Synthesis of current knowledge and a way forward. Glob. Change Biol..

[16] Gaillardet J, Calmels D, Romero-Mujalli G, Zakharova E, Hartmann J (2019). Global climate control on carbonate weathering intensity. Chem. Geol..

[17] Lechuga-Crespo JL (2020). A model to evaluate chemical weathering from riverine transports of dissolved major elements at global scale. Global Planet. Change..

[18] Roelandt C, Goddéris Y, Bonnet M-P, Sondag F (2010). Coupled modeling of biospheric and chemical weathering processes at the continental scale. Global Biogeochem. Cycles..

[19] Bao C, Li L, Shi Y, Duffy C (2017). Understanding watershed hydrogeochemistry: 1. Development of RT-Flux-PIHM. Water Resour. Res..

[20] Li L (2017). Expanding the role of reactive transport models in critical zone processes. Earth Sci. Rev..

[21] Amiotte Suchet P, Probst JL (1995). A global model for present-day atmospheric/soil CO2 consumption by chemical erosion of continental rocks (GEM-CO2). Tellus B.

[22] Aumont O (2001). Riverine-driven interhemispheric transport of carbon. Global Biogeochem. Cycles.

[23] McClain M, Boyer E, Dent C (2003). Biogeochemical hot spots and hot moments at the interface of terrestrial and aquatic ecosystems. Ecosystems.

[24] Berner RA, Lasaga AC, Garrels RM (1983). The carbonate-silicate geochemical cycle and its effect on atmospheric carbon dioxide over the past 100 million years. Am. J. Sci..

[25] Meybeck M (1987). Global chemical weathering of surficial rocks estimated from river dissolved loads. Am. J. Sci..

[26] Munhoven G (2002). Glacial-interglacial changes of continental weathering: Estimates of the related CO2 and HCO3 flux variations and their uncertainties. Global Planet. Change.

[27] Probst, J. L. *Géochimie et hydrologie de l'érosion continentale. Mécanismes, bilan global actuel et fluctuations au cours des 500 derniers millions d'années*. (Université Louis-Pasteur, 1992).

[28] van Vuuren DP (2011). The representative concentration pathways: An overview. Clim. Change.

[29] Moquet J-S (2016). Amazon River dissolved load: Temporal dynamics and annual budget from the Andes to the ocean. Environ. Sci. Pollut. Res. Int..

[30] Mortatti J, Probst J-L (2003). Silicate rock weathering and atmospheric/soil CO2 uptake in the Amazon basin estimated from river water geochemistry: Seasonal and spatial variations. Chem. Geol..

[31] Boeglin JL, Probst JL (1998). Physical and chemical weathering rates and CO2 consumption in a tropical lateritic environment: The upper Niger basin. Chem. Geol..

[32] Donnini M (2016). Chemical weathering and consumption of atmospheric carbon dioxide in the Alpine region. Global Planet. Change.

[33] Moosdorf N, Hartmann J, Lauerwald R, Hagedorn B, Kempe S (2011). Atmospheric CO2 consumption by chemical weathering in North America. Geochim. Cosmochim. Acta.

[34] van Vliet MTH (2013). Global river discharge and water temperature under climate change. Global Environ. Change.

[35] Beck HE (2018). Present and future Köppen-Geiger climate classification maps at 1-km resolution. Sci. Data.

[36] van Vliet MTH, Wiberg D, Leduc S, Riahi K (2016). Power-generation system vulnerability and adaptation to changes in climate and water resources. Nat. Clim. Change.

[37] Amiotte Suchet P, Probst JL (1996). Origines du carbone inorganique dissous dans les eaux de la Garonne. Variations saisonnières et interannuelles/Sources of dissolved inorganic carbon in the Garonne river water. Seasonal and inter annual variations. sgeol.

[38] Streletskiy D, Anisimov O, Vasiliev A (2015). Permafrost Degrad..

[39] Millot R, Gaillardet J, Dupré B, Allègre CJ (2002). The global control of silicate weathering rates and the coupling with physical erosion: New insights from rivers of the Canadian Shield. Earth Planet. Sci. Lett..

[40] Beaulieu E (2010). Impact of atmospheric CO2 levels on continental silicate weathering. Geochem. Geophys. Geosyst..

[41] Lechuga-Crespo JL, Sauvage S, Ruiz-Romera E, George C, Sánchez-Pérez JM (2021). SWATLitho: A hydrogeochemical model to estimate daily geochemical loads at the catchment scale. Environ. Model. Softw..

[42] Delworth TL (2006). GFDL's CM2 global coupled climate models. Part I: Formulation and simulation characteristics. Am. Meteorol. Soc..

[43] Donner LJ (2011). The dynamical core, physical parameterizations, and basic simulation characteristics of the atmospheric component AM3 of the GFDL global coupled model CM3. J. Clim..

[44] Collins WJ (2011). Development and evaluation of an Earth-system model – HadGEM2. Geosci. Model Dev. Discuss..

[45] Martin GM (2011). The HadGEM2 family of met office unified model climate configurations. Geosci. Model Dev..

[46] Dufresne J-L (2013). Climate change projections using the IPSL-CM5 Earth System Model: from CMIP3 to CMIP5. Clim. Dyn..

[47] Watanabe S (2011). MIROC-ESM 2010: Model description and basic results of CMIP5-20c3m experiments. Geosci. Model Dev..

[48] Iversen T (2013). The Norwegian earth system model, NorESM1-M-Part 2: Climate response and scenario projections. Geosci. Model Dev..

[49] Lehner, B. & Grill, G. Global river hydrography and network routing: Baseline data and new approaches to study the world's large river systems. *Hydrol. Process.* **27**, 2171–2186. https://www.hydrosheds.org (2013).

[50] Hartmann J, Moosdorf N (2012). The new global lithological map database GLiM: A representation of rock properties at the Earth surface. Geochem. Geophys. Geosyst..

[51] FAO, IIASA, ISRIC, ISS-CAS & JRC. *Harmonized World Soil Database (version 1.2)*. (FAO, 2012).

[52] Eckhardt K (2005). How to construct recursive digital filters for baseflow separation. Hydrol. Process..

[53] Xie J (2020). Evaluation of typical methods for baseflow separation in the contiguous United States. J. Hydrol..

[54] Amiotte Suchet, P. *Cycle du Carbone, Érosion Chimique des Continents et Transferts vers les Océans*. (Université Louis-Pasteur, 1995).

