# Supplementary material for: Global carbon sequestration through continental chemical weathering in a climatic change context
Source: Sci Rep. 2021 Dec 8;11:23588. doi: 10.1038/s41598-021-02891-y (PMC8654838; doi:10.1038/s41598-021-02891-y)
Supplement: Supplementary file 1 — Supplementary Information. [file 41598_2021_2891_MOESM1_ESM.docx]

**EXTENDED DATA**


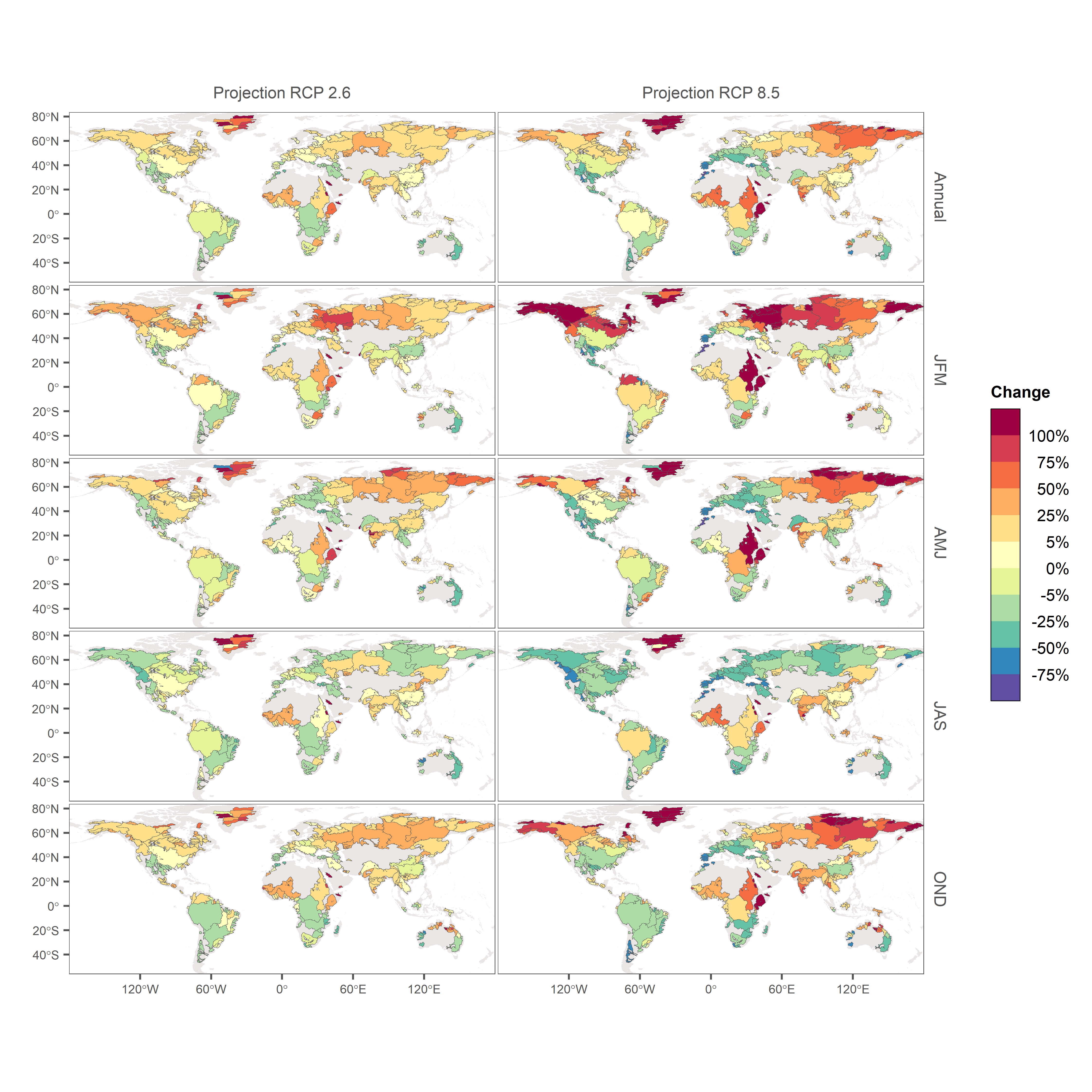


**Extended Data Figure 1.** Projected relative changes (ΔCO_2_, %) in carbon sequestration through chemical weathering of continental rocks for the 300 largest river basins included in the analysis for 2069-2099. Relative changes are computed as the mean value of five simulations, each simulation with each different GCM results for the two scenarios (RCP 2.6 and RCP 8.5). Negative values imply a lower sequestration in the ECP period, while positive values suggest an increased sequestration for the forecasted period. Periods are Annual, January-February-March (JFM), April-May-June (AMJ), July-August-September (JAS) and October-November-December (OND). The maps were created with the R software (version 4.0.3; https://www.r-project.org/).


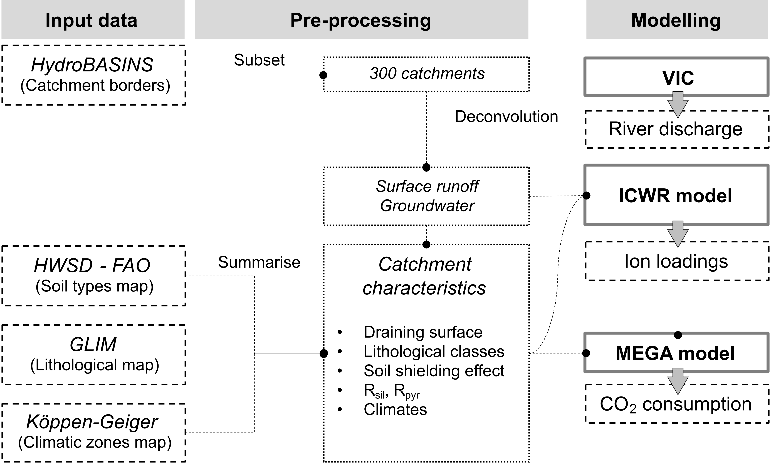


**Extended Data Figure 2**. Workflow summary of the modelling-cascade approach. Input data includes the VIC database^36^, the HydroBASINS^49^, HWSD^51^, GLIM^50^, and the Köppen-Geiger climate classification^35^. The subset is based on the draining area, deconvolution is performed following the Eckhardt^52^ method, and summarisation is accomplished for each catchment by computing the relative area of the soil types, lithological classes, and climatic zones. The VIC model estimates riverine discharges at a global scale^36^. The ICWR model evaluates the ionic fluxes derived from chemical weathering of rocks^36^, the MEGA model balances the ionic loadings to the carbon uptake derived from chemical weathering^32,37,54^.


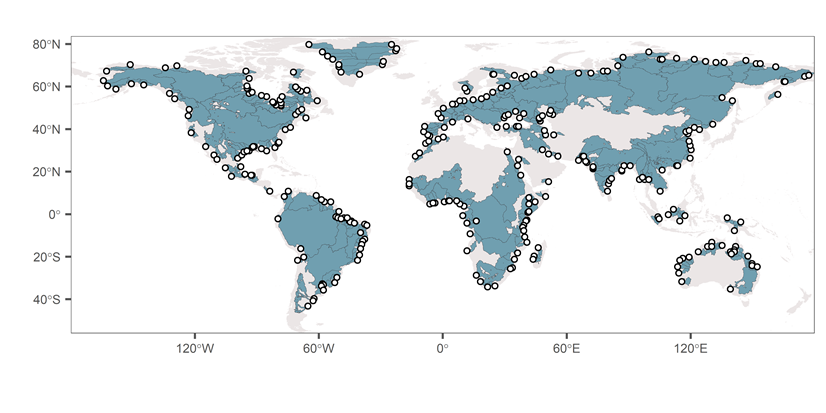


**Extended Data Figure 3**. Map of selected river basins (drainage areas in blue), respective outlets (white circles). The map was created with the R software (version 4.0.3; https://www.r-project.org/).


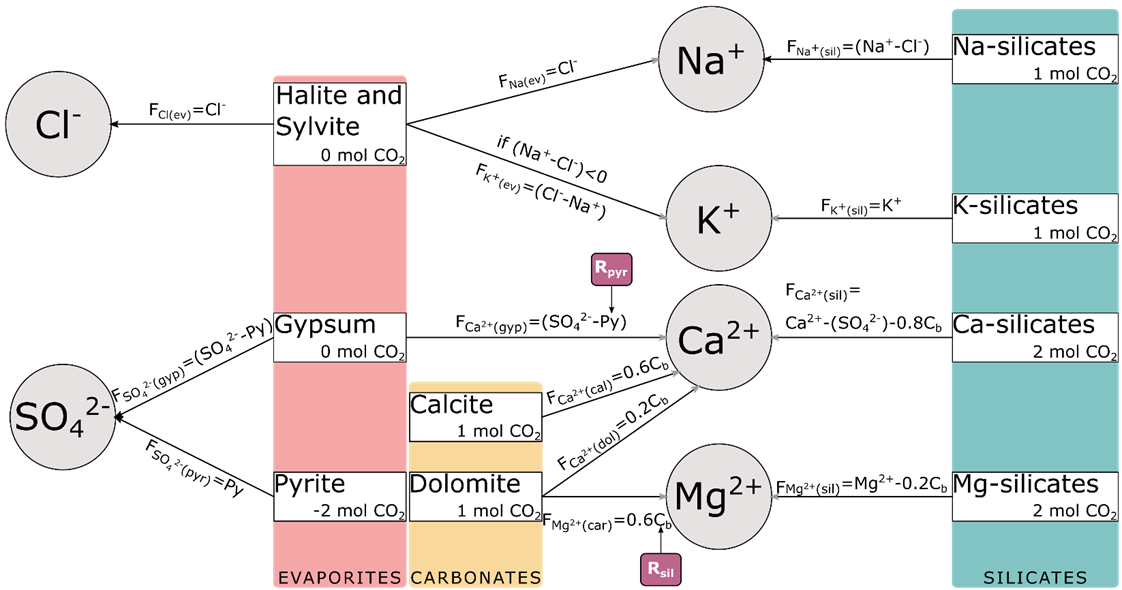


**Extended Data Figure 4**. Mass balance followed in the MEGA model adapted from Amiotte Suchet^54^. Carbon consumption by evaporites, carbonates, and silicates is estimated from a mass balance among the major ion composition found in river water. Further description in Amiotte Suchet and Probst^37^ and in Donnini et al.^32^.

**Extended Data Table 1**. *R_sil_* and *R_pyr_* molar ratios were calculated for each lithological class and compared with Amiotte Suchet and Probst^37^. *R_pyr_** relates to the actual values selected due to the worse representation of the SO_4_^2-^ in the ICWR model, these are taken from Amiotte Suchet and Probst^37^.

| **Rock types** | **Source** | **R_sil_** | **R_pyr_** | **R_pyr_^*^** |
| --- | --- | --- | --- | --- |
| Plutonic and metamorphics | Amiotte Suchet and Probst^37^ | 1,50 | 0,15 |  |
| Volcanic acid |  | 1,20 | 0,02 |  |
| Basalt |  | 0,50 | 0,02 |  |
| Sand and sandstones |  | 1,30 | 0,24 |  |
| Clay detrital rocks (Shales) |  | 0,50 | 0,19 |  |
| Evaporitic | Lechuga-Crespo et al.^17^ |  |  |  |
| Metamorphics |  | 0,23 | 0,26 | 0,15 |
| Plutonic Acid |  | 0,97 | 0,00 | 0,15 |
| Plutonic Basic |  | 4,67 | 0,00 | 0,15 |
| Plutonic Intermediate |  | 3,06 | 0,08 | 0,15 |
| Pyroclastics |  | 1,16 | 0,07 | 0,02 |
| Sedimentary carbonates |  |  |  |  |
| Sedimentary mixed |  | 0,35 | 0,34 | 0,19 |
| Sedimentary siliciclastics |  | 1,13 | 0,13 | 0,24 |
| Sedimentary unconsolidated |  | 1,10 | 0,11 | 0,24 |
| Volcanic acid |  | 1,26 | 0,17 | 0,02 |
| Volcanic basic |  | 0,40 | 0,30 | 0,02 |
| Volcanic intermediate |  | 0,25 | 0,48 | 0,02 |
| R_pyr_ (mol·mol^-1^)= SO_4_^2-^/(Na^+^+K^+^+Ca^2+^+Mg^2+^) and R_sil_ (mol·mol^-1^)= (Na^+^+K^+^)/(Ca^2+^+Mg^2+^) for water draining silicate rocks. R_pyr_* are taken from Amiotte Suchet and Probst^37^ considering the minerals corresponding to each lithological group^50^. | | | | |


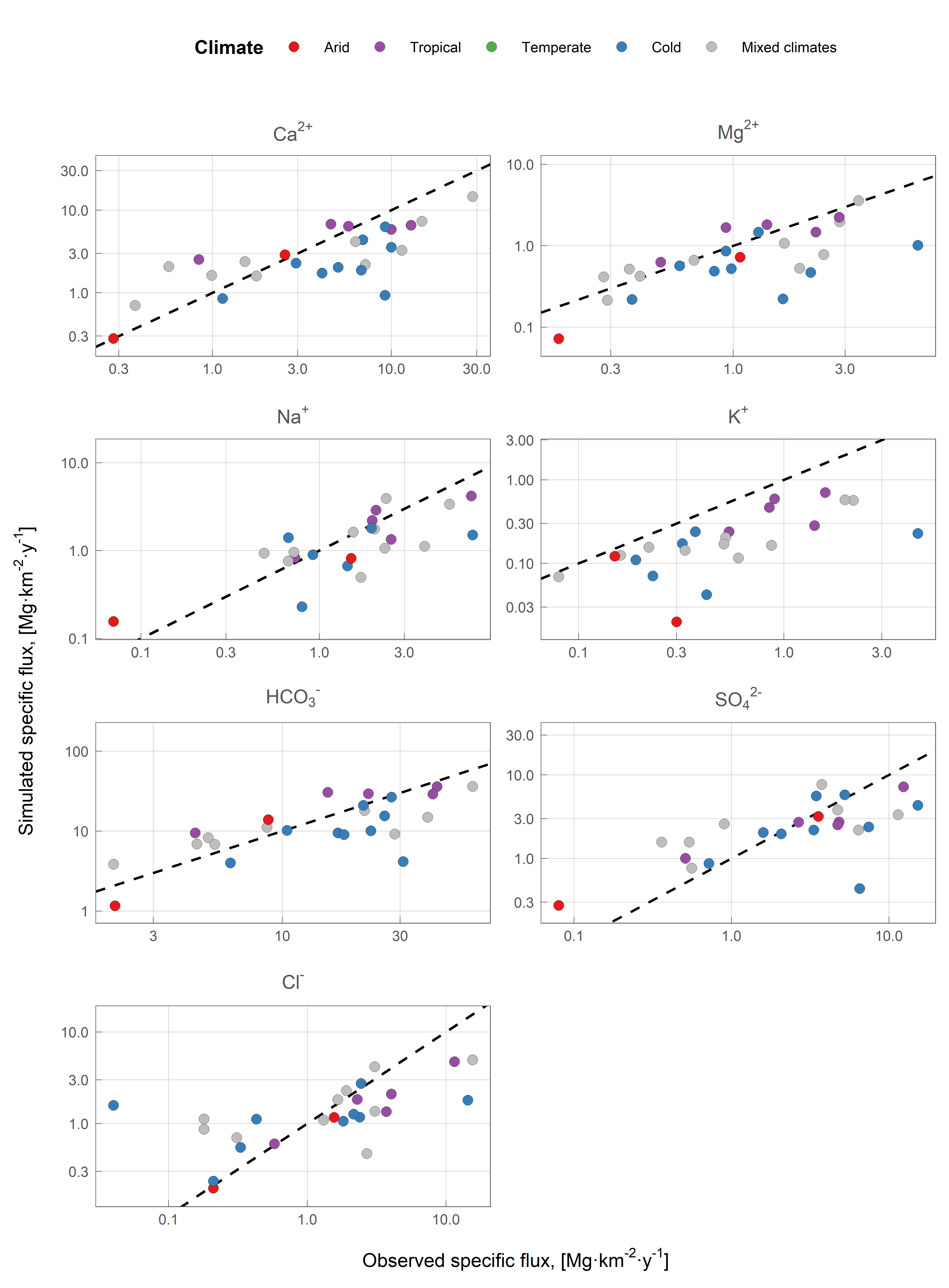


**Extended Data Figure 5.** Evaluation of individual specific ionic river fluxes derived from chemical weathering of rocks in large basins retrieved from Probst^27^. Axes are in logarithmic scale due to the non-normal distribution of the samples, for better appreciation of the correlation. Perfect fit is represented as the dashed line.


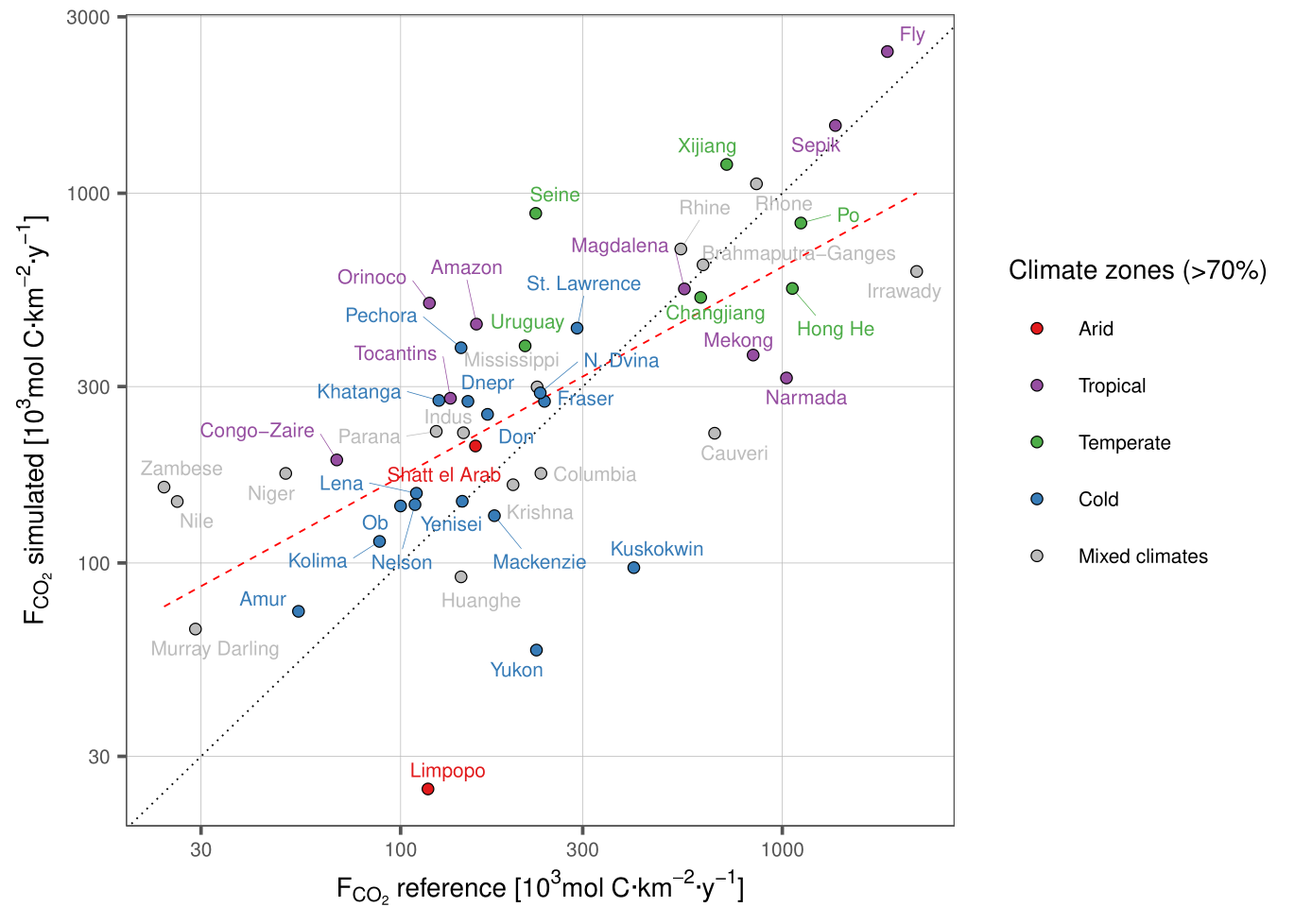


**Extended Data Figure 6.** Comparison of annual simulated CO_2_ fluxes consumed by rock weathering (in the Historical period, 1969-1999) with those derived from field studies, retrieved from Gaillardet et al.^6^. Dashed black line represents perfect fit line. Colours represent predominant current climatic zones, according to Köppen-Geiger classification presented by Beck et al.^35^ while grey dots are those basins with mixed climates. Axis are displayed at the logarithmic scale.
